# A Study on Parametric Amplification in a Piezoelectric MEMS Device

**DOI:** 10.3390/mi10010019

**Published:** 2018-12-29

**Authors:** Miguel Gonzalez, Yoonseok Lee

**Affiliations:** 1 Aramco Research Center–Houston, Aramco Services Company, Houston, TX 77084, USA; 2 Department of Physics, University of Florida, Gainesville, FL 32611, USA; yoonslee@phys.ufl.edu

**Keywords:** MEMS, parametric resonance, nonlinear systems

## Abstract

In various applications, damping from the surrounding fluid severely degrades the performance of micro-electro-mechanical systems (MEMS). In this paper, mechanical amplification through parametric resonance was investigated in a piezoelectrically actuated MEMS to overcome the effects of damping. The device was fabricated using the PiezoMUMPS process, which is based on a Silicon-on-Insulator (SOI) process with an additional aluminum nitride (AlN) layer. Here, a double-clamped cantilever beam with a concentrated mass at the center was excited at its first resonance mode (out-of-plane motion) in air and at atmospheric conditions. A parametric signal modulating the stiffness of the beam was added at twice the frequency of the excitation signal, which was swept through the resonance frequency of the mode. The displacement at the center of the device was detected optically. A four-fold increase in the quality-factor, *Q*, of the resonator was obtained at the highest values in amplitude used for the parametric excitation. The spring modulation constant was obtained from the effective quality-factor, $Q_{eff}$, versus parametric excitation voltage curve. This study demonstrates that through these methods, significant improvements in performance of MEMS in fluids can be obtained, even for devices fabricated using standard commercial processes.

## 1. Introduction

Micro-electro-mechanical systems (MEMS) have enabled the miniaturization of a wide variety of sensor elements, allowing their integration into ever-smaller sensing platforms accross industries. Likewise, their small size and mass makes them very sensitive to weak forces that change their resonant properties. However, the small inertia associated with their motion also makes MEMS very susceptible to dissipative losses from the fluidic medium in which they are operated. While this shortcoming may be avoided through packaging and optimal device design in several applications (such as in accelerometers, gyroscopes, etc.), there are fewer viable solutions when the device needs to be exposed to the gas or liquid, such as in biological or environmental sensing. Therefore, an efficient “damping control” system in MEMS and nano-electro-mechanical systems (NEMS) may improve their performance for applications such as high-resolution viscometry and densitometry, mass and chemical sensing, and flow sensing, for both industrial applications [1,2,3,4,5] and the study of fundamental fluid mechanics and condensed matter systems at the micro and nanometer scales [6,7,8,9,10,11].

While electronic feedback and signal control techniques have been used for dissipation control [12], a less resource demanding solution could potentially be attained through mechanical amplification via parametric resonance [13,14]. This effect occurs in a resonant system when a time varying parameter is modulated, typically at twice the resonant frequency of the system. Its most familiar manifestation occurs while propelling ourselves in a swing at the park, where by modulation of our mass distribution we are able to propel ourselves to larger heights. In various systems, the most commonly modulated parameter is the elasticity, so the standard damped and forced harmonic oscillator equation becomes

(1) $$m\ddot{z}+\gamma \dot{z}+\left[k_{0}+\delta k\left(t\right)\right]z=F\left(t\right),$$

where $\delta k\left(t\right)=\kappa \sin\left(\omega_{p}t\right)$ represents a periodic modulation of the spring constat, $\omega_{p}=2\omega_{0}$, and where $\omega_{0}=\sqrt{k_{0}/m}$ is the resonance frequency of the system and $\omega_{p}$ is the frequency of the parametric signal. While Equation (1) is a linear equation, it does not have a known closed form solution. However, approximate solutions can be obtained by perturbative and other techniques, such as Floquet theory [15]. The amplitude of the resulting motion, z(t), is dependent upon the modulation amplitude, κ, and the phase angle, ϕ, of the excitation F(t) with the elasticity modulation. In general, the parametric mechanical gain can be written as [16]

(2) $$G\left(\phi \right)={\left[\frac{\cos^{2}\left(\phi +\pi /4\right)}{{\left(1+Q\kappa /2k_{0}\right)}^{2}}+\frac{\sin^{2}\left(\phi +\pi /4\right)}{{\left(1-Q\kappa /2k_{0}\right)}^{2}}\right]}^{1/2},$$

where $Q=\frac{m\omega_{0}}{\gamma }$ is the quality factor.

Parametric resonance in microsystems was first reported by Rugar and Grüter [16]. It has also been considerably studied in NEMS [17], and its properties have been exploited in high-sensitivity nanoscale imaging in scanning probe microscopy (SPM) [18], atomic force microscopy (AFM) [19], and in novel ultra-sensitive force and mass sensing techniques [20,21]. While most demonstrations of parametric resonance in microsystems usually make use of capacitive actuation techniques [22,23], since the electrostatic spring effect is a simple parameter to quantify and control, it has also been demonstrated recently in fully piezoelectric devices by modulation of the elasticity through the variable tension forces from the motion of a vibrating beam in a double-clamped geometry [24,25,26,27].

Here, we demonstrate parametric resonance on a piezoelectric MEMS device fabricated using a multi-user shared wafer MEMS prototyping process. We evaluate the increase in effective quality-factor, $Q_{eff}$, as a function of the parametric pump signal and obtain the spring modulation constant from direct fit to the theory. Significant increases in $Q_{eff}$ were obtained, suggesting that parametric amplification could be a useful tool to improve the performance of MEMS when operated in fluids.

## 2. Materials and Methods

### 2.1. MEMS Device

The device was fabricated using the PiezoMUMPS fabrication process by MEMSCAP (Durham, NC, USA) [28]. This process starts with a Silicon-on-Insulator wafer as the substrate. A 10 μm doped Silicon layer provides the structural material. A 0.5 μm piezoelectric aluminum nitride (AlN) layer is deposited over the wafers using reactive sputtering. Both layers are patterned using standard lithographic techniques and the device is mechanically released by back-etching a trench under the moving structures.

**Figure 1:**
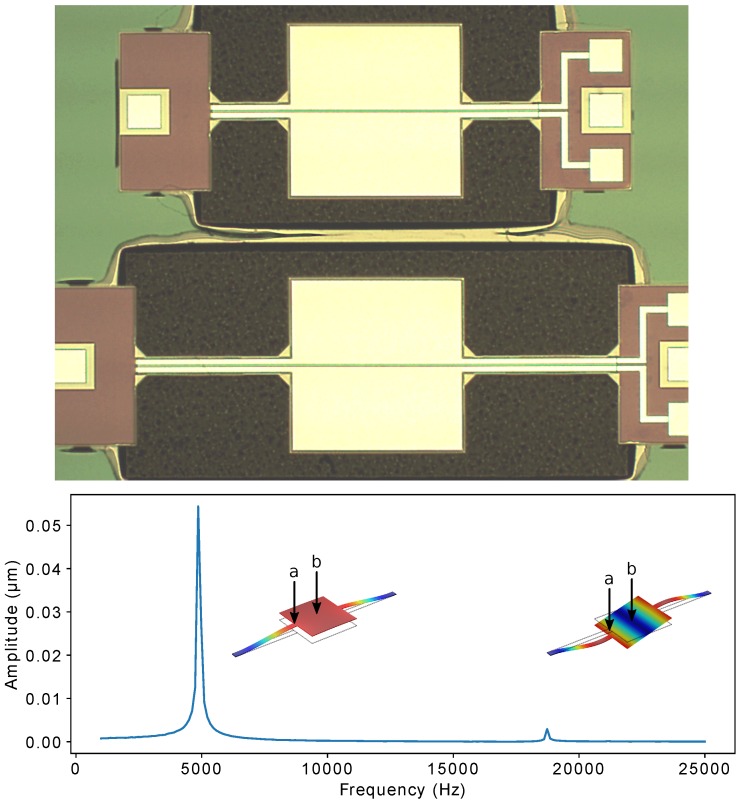
**Top**: Optical image of typical devices with different supporting beam lengths. The device used has 1050 μm length beams supporting an 800 μm square plate. **Bottom**: Coarse frequency sweep showing the two main modes. Displayed modeshapes were obtained from COMSOL simulations. Points “a” and “b” denote the location of the laser beam spot for the laser doppler vibrometer (LDV). The color code on the modeshapes indicates red for areas of maximum displacement and blue for no displacement.

In Figure 1, an optical image of a typical pair of fabricated devices is shown. The device used in this work consists of a pair of support beams with dimensions of 1050 μm × 90 μm and a center square plate with side length of 800 μm. A piezoelectric AlN layer covers the entire moving structure, while separate metal layers cover symmetrically each half of the device in order to get separate actuation and detection electrodes. Alternatively, as was done throughout this work, both electrodes can be used to drive the device for optical detection.

COMSOL simulations for the eigenfrequencies of the first two vibrational modes gave values of 5621 Hz and 20,541 Hz, respectively. The modeshapes are shown at the bottom of Figure 1, where a coarse frequency sweep through both modes is shown. The sweep was performed using both electrodes to actuate the device symmetrically in the out-of-plane direction. First, in order to measure the displacement from both modes, the laser spot from a laser doppler vibrometer (LDV) was focused at the junction between one of the support beams and the center plate (point “a” in Figure 1). Next, the spot was moved towards the center of the plate (point “b” in Figure 1), where only the first mode was visible, as expected. The laser spot was kept at the center location for subsequent measurements of the first mode using the parametric drive.

### 2.2. Measurement Scheme

**Figure 2:**
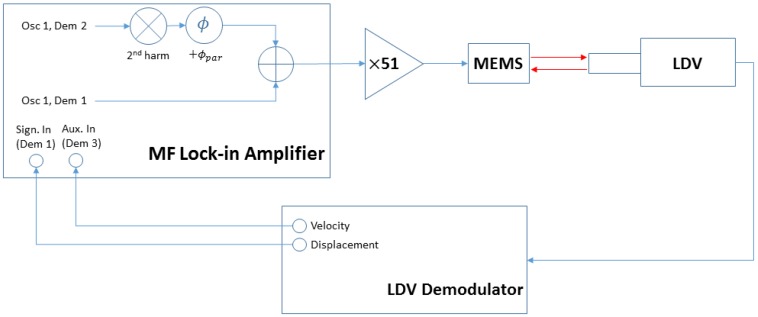
Schematic diagram for measurement. A multi-frequency lock-in amplifier is used to generate the output signal to the micro-electro-mechanical systems (MEMS) and to detect the displacement and velocity signals from the LDV.

A schematic diagram of the measurement is shown in Figure 2. A multi-frequency lock-in amplifier (MFLI, Zurich Instruments, Zurich, Switzerland) was used to generate the input signal to the MEMS and to detect the displacement and velocity signals from the LDV. The second harmonic of the excitation, provided by the lock-in oscillator 1 (Osc 1 in Figure 2), was added to the output signal, so that the first harmonic can be used as the main excitation signal and the second harmonic as the parametric drive. The amplitude of the first harmonic was kept at 2 mV${}_{pp}$ in the MFLI. The amplitude of the parametric drive was swept through various values, as explained below. The phase of the parametric signal was controlled by the internal phase shifter of the lock-in prior to being added to the output. The output was then amplified with a constant voltage gain of 51 by a power amplifier (TEGAM 2350, TEGAM, Geneva, OH, USA) and then applied symetrically to the two electrodes covering both halves of the device. The motion of the center plate of the device was detected by an LDV system (OFV-534/OFV-5000, Polytec GmbH, Waldbronn, Germany). A 25× microscope objective was used to focus the laser at the center of the plate. The sensitivities of displacement and velocity LDV demodulators were kept at 1 μm/V and 50 mm/s/V, respectively. The outputs from the demodulators were then fed back to the lock-in amplifier into demodulators 1 and 3 (Dem 1 and Dem 3). The demolutator 2 block (Dem 2) was only used to obtain the parametric output from its reference path.

## 3. Results

**Figure 3:**
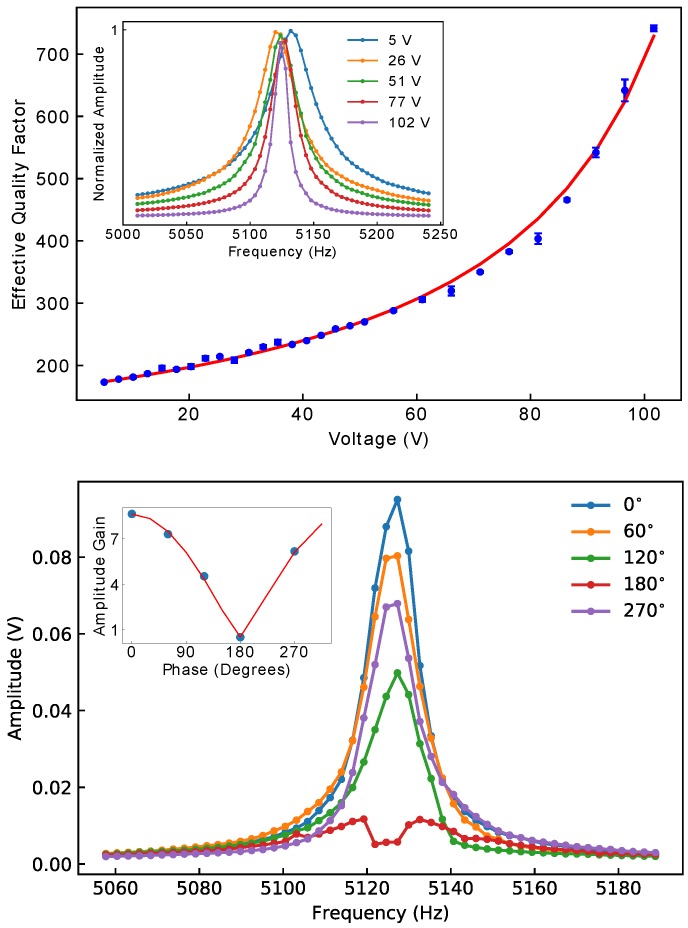
**Top**: Effective quality-factor $Q_{eff}$ as a function of parametric excitation. The solid red line is a fit to Equation (3). The inset shows examples or resonance peaks at different voltages. The amplitude of the peaks was normalized by the fitted corresponding amplitude of each peak. **Bottom**: Resonance peaks as the phase of the parametric signal was varied while the parametric excitation voltage was kept at 102 V. The inset shows the amplitude gain as a function of phase. The red line was fitted from Equation (2).

Frequency sweeps of the excitation signal (first harmonic) were performed at various amplitudes of the parametric drive (second harmonic), from 5 V to 102 V${}_{pp}$. The parametric drive was locked at twice the excitation frequency. The excitation amplitude was kept at 2 mV${}_{pp}$ (102 mV${}_{pp}$ after amplification by the output amplifier) and the phase shifter at the MFLI was kept at zero for both the parametric and the excitation drives. The quality factor, *Q*, increased monotonically as the parametric drive amplitude was increased, as shown in the top plot in Figure 3. The inset shows the resonance peaks obtained from the displacement measurement (Dem 1). The amplitude of the peaks was normalized by the peak amplitude obtained from fits to the lorentzian lineshapes. At the highest excitation, a four-fold increase in quality-factor was obtained. The solid line in the inset of the top of Figure 3 is a fit to the model of Equation (3), where the effective quality factor, $Q_{eff}$, at maximum gain phase was written as

(3) $$\frac{Q_{eff}}{Q}=\frac{1}{1-\left(\frac{V}{V_{e}}\right)},$$

and where the spring modulation was assumed proportional to the parametric voltage excitation ($\kappa =\tilde{\kappa }V$). Linearity on amplitude versus direct drive force was checked on various devices. A Duffing nonlinearity was consistently seen at displacements close to 1 μm, much larger than those in these experiments. The single fitting coefficient, $V_{e}$, was defined as $1/V_{e}=Q\tilde{\kappa }/2k_{0}$. From the obtained fitted parameter value, $V_{e}=131.9$ V, we estimated the modulation intensity, $\tilde{\kappa }$, using the value of $k_{0}=19.1$ N/m, obtained from the measured frequency value $f_{0}$ and the calculated mass value $m=1.8\times 10^{-8}$ Kg, and using the initial quality factor Q=167. The center mass of the device was obtained from the known geometry and the densities of silicon, aluminum (metal), and aluminum nitride (piezo). The obtained value of the modulation intensity was $\tilde{\kappa }=1.81\times 10^{-3}$ N/m/V. Comparing with Ref. [25], the fitted values obtained at the largest parametric excitations ($V/V_{e}=0.77$) correspond to a modulation below the linear instability point ($V/V_{e}\to 1$). Also notice that, as in Ref. [25], the effective quality-factor and the gain are not trivially related, however, both are function of the dimensionless parametric drive $V/V_{e}$.

The gain is also dependent upon the relative phase between the drive and the parametric signal. As shown at the bottom of Figure 3, resonance curves were obtained from frequency sweeps as the phase of the parametric drive was varied and the parametric excitation voltage was kept at the maximum value (102 V${}_{pp}$). The resonance was *quenched* at $\phi_{p}=180^{{}^{\circ}}$. Note that in this experiment we varied the parametric signal phase (see Dem 2 in Figure 2), which changes by half of the phase of the direct drive (Dem 1). Accounting for an additional $45^{{}^{\circ}}$ phase lag, peak quenching is then predicted to occur at $180^{{}^{\circ}}$, as was observed in the experiment. Similarly, the $45^{{}^{\circ}}$ phase lag predicts maximum amplification at phase angle $0^{{}^{\circ}}$ in Dem 2. The solid line in the inset at the bottom of Figure 3 is a fit to Equation (2), including the aforementioned phase considerations. The gain was obtained from the ratio of the amplitude at the peak center with the parametric pump signal and the peak amplitude with no pump signal. The value of $V_{e}$ obtained from the fit was 115.4 V, in close agreement with that obtained from the effective quality-factor. At lower gains the lineshape becomes asymmetric and eventually a dip at the middle of the peak is formed. At this point, maximum de-amplification occurs and the gain tends to 0.5, as predicted by the theory. The lineshape of the displacement resonance curves obtained are of the same shape and follows a similar trend as in Ref. [25] and as the theoretical response from Ref. [29] below the linear parametric instability point.

## 4. Discussion

A study on parametric resonance in a piezoelectric MEMS device was presented. The device was fabricated using the PiezoMUMPS process by MEMSCAP. Parametric amplification was evaluated in this device when operated in air and at atmospheric conditions. A four-fold increase in the quality-factor was obtained at the highest parametric excitations used. The spring constant modulation parameter was obtained from the theory. While better amplification can be found in other devices studied in the literature, the devices presented here, fabricated using a simpler commercial process, display enough of an effect such that their performance can be significantly improved for applications where large damping effects need to be overcome.

Future work could be focused on improving the parametric response by optimizing the device design so that lower modulation voltages can be used. Also, parametric resonance of MEMS in liquids, where much larger damping effects are expected, has not been given a greater amount of attention to the best of the authors’ knowledge. A systematic study on the effects of damping on the parametric instability point could be performed by sweeping the device response at difference parametric excitations and mapping out the changes in the linear instability tongue as the viscosity of the liquid is varied. In the linear instability region, large changes in the dynamical state of the device are observed from smaller external perturbations, opening the door to enhanced sensitivity detection in various technological applications [20,21].

The workflow presented in this paper provides a self-contained protocol to test the efficiency of parametric amplification in piezoelectric MEMS where the spring modulation is attained through geometric effects. Ultimately, this could enable improved usage of these devices in highly damped environments such as in gases and liquids and for applications in viscometry and fluid analysis as well as for testing fundamental fluid mechanics at the micro and nanometer scales.
